# The prevalence of teenage pregnancy and early motherhood and its associated factors among late adolescent (15–19) years girls in the Gambia: based on 2019/20 Gambian demographic and health survey data

**DOI:** 10.1186/s12889-022-14167-9

**Published:** 2022-09-17

**Authors:** Bewuketu Terefe

**Affiliations:** Department of Community Health Nursing, School of Nursing, College of Medicine and Health Science, University of Gondar, Gondar, Amhara Ethiopia

**Keywords:** Pregnancy and early motherhood, Late Adolescent, Determinants, Gambia

## Abstract

**Introduction:**

Pregnancy and early motherhood among teenage girls is the current issue of public health burden in developing countries. Although the Gambia has one of the highest adolescent fertility rates in Africa, there is no data record about it in The Gambia. Therefore, this study aimed to assess the prevalence of pregnancy and early motherhood and its determinants among late adolescent girls in the Gambia.

**Methods:**

It is a secondary data analysis using the 2019–20 Gambian demographic and health survey data. A total of 2,633 weighted 15–19 years old girls were included in the study. Using Stata 14 version, a pseudo logistic regression analysis method was employed to declare factors significantly associated with pregnancy and early motherhood among 15–19 years old late-adolescent girls in the Gambia. Variables with a *p*-value of < 0.2 were entered into multivariable regression analysis, and after controlling other confounding factors adjusted odds ratio of 95% CI was applied to identify associated variables.

**Results:**

Pregnancy and early motherhood were found in 13.42% of late adolescent Gambian girls. Logistic regression analysis depicted that a unit increase in adolescent age was positively significantly associated with pregnancy and early motherhood (adjusted odds ratio [aOR] = 2.15; 95% confidence interval [CI] = 1.93,2.39), after period ended knowledge of ovulatory cycle (aOR = 1.99; 95% CI = 1.23,3.22), being from a family size of greater than ten (aOR = 1.25; 95 CI = 1.01,1.55) times more likely to become pregnant and early motherhood than their counterparts respectively. In contrast, rich in wealth (aOR = 0.35; 95% CI = 0.23,0.54), having primary education (aOR = 0.58; 95% CI = 0.43,0.79), secondary and above education (aOR = 0.12; 95% CI = 0.09,0.17).

**Conclusion:**

Pregnancy and early motherhood remain significant public health challenges in the Gambia. Strengthening female education, empowerment, reproductive health life skill training and awareness, encouraging disadvantaged females, and designing timely policies and interventions are urgently needed.

## Introduction

Only in developing regions did around 12 million girls aged 15–19 years become pregnant, and a minimum of 777,000 of them under 15 years gave birth with more than 10 million unintended pregnancies, 3.9 million unsafe abortions, and other related pregnancy complication-induced mortality and morbidity per a year [1–3]. Although early pregnancy is a global public challenge, it is more severe and mainly occurs with many other related problems in developing nations [4]. The scope of the problem has tremendous variations across regions, from 0.3%, 33%, and 83% in Korea, South-East Asia, and Bangladesh, respectively, since 2018 [5, 6]. Early pregnancies have enormous health consequences for both mothers and their babies. In middle- and low-income nations, early pregnancy takes up 99% of death of 15–49 years mothers [3]. Mothers having early pregnancy have a higher chance of exposure to eclampsia, systemic infections, preterm, and low birth weight than their elders [7]. The consequences of early pregnancies do not end with their health, but also it has a damaging result on their social and economic status. Girls who became pregnant before their 18 years also will face violence, stigma, dropping out of school, and employment opportunities [8, 9].

Early pregnancy and motherhood remain a tremendous public health concern. Millions of girls and their babies suffer from early and unintended pregnancies and complications [10]. Early and unintended pregnancies are major public issues globally; nevertheless, the challenge is more severe and consistent in developing countries, with an enormous variety of rates. Some African countries' reports depicted that the overall rate of early pregnancy was 18% in Kenya, 29% in Malawi and Zambia, and Ethiopia, ranging from 3 to 23% in different regional states [10, 11]. Other multicountry analyses in sub-Saharan revealed 44.3% in Congo, 36.5% in Rwanda, and 75.6% in Chad [12]. Several studies show many factors contribute to early and unintended pregnancies and their complications in Sub-Saharan countries. For illustration, among individual factors attributed to early pregnancies, use of alcohol, level of education, curiosity, and low self-esteem were factors significantly associated with early pregnancies and their complications [10, 12–14]. Health service-related factors such as the cost of contraceptives, lack of privacy at health facilities, lack of sexual education, unskilled health providers, negative attitudes toward health prodders to deliver reproductive health services, and non-friendly adolescent reproductive services also contributed to a negative impact on early and unintended pregnancies [13–16]. Social-cultural factors such as peer influence, coercive sexual relations, unequal gender power, absence of free education, early marriage, religion, poverty, and lack of parental counselling were other predictors of early and unintended pregnancies [13, 15, 17–21].

Studies done in the west, and central African nations, including the Gambia, have come with a high rate of adolescents maternity, up to 49% in the central African republic to 16% in Senegal, and early marriage variations from 61% in Niger to 6% in Ghana [22]. Although substantial studies did not conduct on pregnancy and early motherhood in the Gambia, few reports indicated that girls marry about ten years earlier than the mean, with significant variation across regions, 9% in Brikama to 29% in Kuntaur, with an overall burden of 14% in the country [23]. About 32% of the population is adolescent; however, only 7 to 9% utilize family planning due to poor quality of care, lack of knowledge, and other cultural constructs [24]. Teenage pregnancy and early motherhood could expose females to infections like HIV, unintended pregnancy, and high maternal mortality. In the Gambia, unintended pregnancy has shown a high rate of increase, from 18,000 in 2012 to 20,000 in 2016 [25]. A study conducted in the Gambia shows 15–19 years old females tend to experience unintended pregnancies more than the orders [26].

The study dramatically benefits rural adolescents who are twice as likely to be married and mothers at risk of maternal mortality twofold as urban mothers and in a state with more than five times the difference in maternal mortality across regions [23, 27]. Although teenage pregnancy is increasing at an alarming rate, there is no systematic program in the Gambia that focuses on the health needs of adolescents and young people, and parents rarely have conversations with their teenage children, especially the girls, about matters of sex or adolescence [28, 29]. Pieces of evidence from the Gambia showed that there is a significant difference in ethnicity, educational status, perception, culture, and with which parents the girl lives [30, 31].

Recently, many African countries, the African Union, world banks, and many other stakeholders have been concerned to draft policies and soft options regarding the steadily increasing rate and complications of early and unintended pregnancies in Africa [32]. This study will have a positive influence on policymakers, planners, health institutions, women's affairs agencies, and other stakeholders to draft an effective policy to halt early and unintended pregnancies; hence many studies recommended that developing countries should develop and implement a multisectoral approach social policies, programs and plans to reduce early pregnancies and complications by increasing health care delivery quality, by empowering adolescent girls in education, family planning and use of modern contraceptive methods [12, 33]. To the best of my searching knowledge, no data is recorded on teenage pregnancy and early motherhood in the Gambia. The study will examine the public burden patterns and pregnancy and early motherhood determinants. The study will provide preliminary information for the upcoming researchers, the Gambia government, the Gambia community, and those stakeholders who are working on women's and children's health to draft policy and implement the procedure accordingly. This study aimed to determine the potential factors of pregnancy and early motherhood among late adolescent girls 15–19 years old in the Gambia.

## Methods and materials

### Study design and setting

This study was based on an extensive nationally representative based survey, Gambia Demographic Health Survey (GDHS), conducted in the Gambia from 21 November 2019 to 30 March 2020. The Gambia is located on the West African coast. It is bordered on the North, South, and East by the Republic of Senegal and on the west by the Atlantic Ocean. The country has a tropical climate characterized by the rainy season (June – October) and the dry season (November–May).

### Source population and sampling technique

The survey employed a stratified two-stage cluster sampling. In the first stage, EAs were selected with a probability proportional to their size within each sampling stratum. In the second stage, the households were systematically sampled. In the first stage, 281 EAs were selected. In the second stage, an average of 25 households were selected per cluster/EA. The data is freely available in public. We accessed the dataset used for the present study after registering and receiving an authentication letter from the Demographic and Health Survey (DHS) program at The DHS Program—Gambia: Standard DHS, 2010–20 Dataset. The source of the population was all late adolescent pregnancy and early motherhood (15 to 19 years) in The Gambia. Late adolescents and early motherhood who were pregnant and/or had a child in the last five years before each survey were the study population. The adolescents’ sample weightings were used in the estimation to provide to overcome disproportional allocations of samples during data collection. Accordingly, 2,633 weighted 15–19 years old adolescent samples were included in the study yielding a response rate of 97%.

### Variables of the study

#### Dependent variables

The main dependent variable of this study was pregnancy and early motherhood among adolescents. The survey was taken as a dependent variable for adolescents [15–19] who had a child or pregnancy in the past five years. The response variable was dichotomized into "late-adolescent pregnancy = 1" and "late-adolescent non-pregnancy = 0". It might be defined as the percentage of Adolescents who became mothers, are pregnant with their first child, and have begun childbearing. These included all girls from the age of 15 to 19 years old at the survey time. The percentage of adolescent women who are mothers was calculated by dividing the number of adolescent women who have had birth by the total number of teenage women, including those without birth. The percentage of pregnant women with the first child was calculated by dividing the number of women who have not had birth but who are pregnant at the time of data collection by the total number of teenage women, including those without birth. The percentage of women who have begun childbearing was calculated by adding the number of women who either have had a birth or are pregnant at the time of the interview and dividing by the total number of teenage women, including those without a birth.

#### Independent variables

The independent variables included: age, educational status, wealth status, occupational status, marital status, knowledge of ovulatory cycle, knowledge of any family planning, relationship with household head, age of the household head, sex of the household head, household family size, own a mobile phone, use of the internet, frequency of watching to television, listening to the radio, reading a magazine, entries to birth history, contraceptive use and intention, region, religion, ethnicity, age at first sex, heard family planning by text messages, heard family planning from health professionals, heard family planning from traditional communicators, heard family planning from friends/relatives, place of residence and health insurance. variables were collected based on previous works of literature and scientific facts related to the outcome variable [34–38].

### Data processing, procedure, and analysis

Data were extracted from individual records (IR) files, and further coding and transformations were done using statistical software, STATA version 14. The weighted samples were utilized for analysis to adjust for unequal probability of selection and non-response in the original survey. Descriptive, summary and analysis statistics were done using STATA version 14 software. The data were weighted using sampling weight, primary sampling unit, and strata before any statistical analysis to restore the survey's representativeness and tell the STATA to consider the sampling design when calculating standard errors to get reliable statistical estimates. Completed DHS questionnaires were carefully coded, entered, and edited after data collection was done [39]. Bivariable logistic regression was used to select candidate variables for multivariable logistic regression. In the Bivariable logistic regression, a *p*-value of less than 0.2 was used as a cut point to choose variables for the multivariable analysis entry. Multivariable logistic regression was used to identify independent predictors of pregnancy and early motherhood in the Gambia, controlling confounders. 95% confidence interval (CI) and *p*-value < 0.05 were used to determine the statistical significance. Using the variance inflation factor, a pseudo linear regression was fitted to assess multicollinearity among the independent variables. Moreover, Hosmer and Lemeshow test was used to evaluate the overall model fitness of the final regression model.

## Result

### Sociodemographic characteristics study population

In this survey, 2,633 weighted samples of late adolescent girls [15–19] were considered for analysis. 530(20.11%) participants were 19 years old. In this study, concerning wealth status, marital status, educational background, and religious affiliation, 1,163(44.15%), 2,136(81.11%), 1,728(65.62%) and 2,556(97.10%) of them were wealthy, single, secondary and above and Islamic affiliation respectively. In the same way of expression, 1,878(71.33%), 220(8.34%), and 2,298(87.27%) of the participants did not have current work, know the ovulatory cycle, and heard about family planning from health professionals, respectively. However, 1,943((73.79%) of them did not have the intention to use the contraceptive method shortly. In place of residence, region, and ethnicity characteristics, 1,901(72.20%), 1,174(44.60%), and 862(32.73%) belonged to urban, Brikama, and Mandinka/Jahanka ethnicities, respectively. All in all, the fee of health care costs were not covered by health insurance (Table 1).

**Table 1:** Sociodemographic characteristics of 15–19 years old late-adolescents who had pregnancy or children in the past five years preceding the survey in the Gambia, GDHS 2019/20 (*n* = 2,633)

Variables	Pregnancy and early motherhood		Total, n (%)
	No, n (%)	Yes, n (%)	
Age			
15	463(99.00)	5(1.00)	468(17.78)
16	544(96.31)	21(3.69)	565(21.46)
17	485(87.96)	66(12.04)	551(20.94)
18	412(79.41)	107(20.59)	519(19.71)
19	375(70.79)	155(29.21)	530(20.11)
Wealth index			
Poor	759(80.00)	190(20.00)	949(36.05)
Middle	432(82.96)	89(17.04)	521(19.79)
Rich	1,088(93.57)	75(6.43)	1,163(44.15)
Marital status			
Single/divorced/widowed	2,074(97.10)	62(2.90)	2,136(81.11)
Married/living together/separately with partners	206(41.39)	291(58.91)	497(18.89)
Educational attainment			
No education	279(64.22)	156(35.78)	435(16.52)
Primary	374(79.52)	96(20.48)	470(17.86)
Secondary and above	1,626(94.13)	102(5.87)	1,728(65.62)
Occupational status			
Currently not working	1,665(88.68)	213(11.32)	1,878(71.33)
Currently working	614(81.35)	141(18.65)	755(28.67)
Religion			
Islamic	2,205(86.27)	351(13.73)	2,556(97.10)
Christian	74(96.76)	3(3.24)	77(2.90)
Ethnicity			
Mandinka/Jahanka	789(91.50)	73(5.80)	862(32.73)
Wollof	300(88.22)	40(11.78)	340(12.92)
Jola/Karoninka	262(94.36)	16(5.64)	277(10.53)
Fula/Tukulur/Lorobo	442(84.53)	81(15.47)	523(19.24)
Serere	86(92.06)	8(7.94)	94(3.56)
Sarahule	173(81.99)	38(18.01)	211(8.02)
Creole/Aku /Marabout	14(100.0)	0(0.0)	14(0.54)
Manjago	25(100.0)	0(0.0)	25(0.93)
Bambara	32(84.86)	6(15.14)	38(1.42)
Other	12(65.75)	7(34.25)	19(0.72)
Non-Gambian	145(62.81)	86(37.19)	231(8.79)
Knowledge of ovulatory cycle			
During her period	90(89.02)	11(10.98)	101(3.85)
After period ended	638(80.33)	156(19.67)	795(30.18)
Middle of the cycle	332(84.51)	61(15.49)	393(14.93)
Before period begin	306(87.90)	42(12.10)	349(13.24)
At anytime	196(89.09)	24(10.91)	220(8.34)
Do not know	717(92.40)	59(7.60)	776(29.46)
Knowledge of any methods			
No know	127(92.52)	10(7.48)	137(5.20)
Knows modern method	2,153(86.25)	343(13.75)	2,496(94.80)
Relationship with the household head			
Head and wife	17(17.89)	75(82.11)	92(3.49)
Daughter	1,079(94.29)	66(5.71)	1,145(43.48)
Daughter-in-law	38(33.12)	76(66.88)	114(4.32)
Granddaughter	165(96.90)	5(3.10)	170(6.47)
Sister and other relatives	694(87.14)	103(12.86)	797(30.26)
Adopted and not related	287(90.88)	29(9.12)	316(11.98(
Age of household head			
18–28	69(66.41)	35(33.59)	104(3.95)
29–39	271(82.31)	58(17.59)	329(12.49)
40–50	614(89.14)	75(10.86)	689(26.15)
51–61	673(90.08)	74)9.92)	748(28.39)
= 62 +	653(85.43)	111(14.57)	764(29.02)
Household member			
1–10	1,055(87.24)	154(12.76)	1,209(45.93)
= 10 +	1,224(86.01)	199(13.99)	1,423(54.07)
Owning mobile phone			
No	1,106(89.20)	134(10.80)	1,240(47.10)
Yes	1,173(84.24)	219(15.76)	1,393(52.90)
Use of the internet			
Never	1,140(88.07)	154(11.93)	1,294(49.15)
Ever use	1,140(85.14)	199(14.86)	1,339(50.85)
Watching to television			
No	958(83.65)	187(16.35)	1,145(43.50)
Yes	1,322(88.83)	166(11.17)	1,488(56.50)
Listening radio			
No	1,584(86.79)	241(13.21)	1,825(69.30)
Yes	696(86.09)	112(13.91)	808(30.70)
Reading to magazine/newspaper			
No	2,204(86.20)	353(13.80)	2,556
Yes	76(99.14)	.7(0.86)	77
Entries in the birth history			
No	2,271(96.50)	82(3.50)	2,353(89.37)
Yes	9(3.14)	271(96.86)	280(10.63)
Contraceptive use and intention			
Not intended	1,727(88.87)	216(11.13)	1,943((73.79)
Using any method	3(7.13)	38(92.87)	41(1.56)
Intended to use	550(84.75)	99(15.25)	649(24.65)
Heard family planning by text message			
No	2,248(86.75)	344(13.25)	2,593(98.43)
Yes	31(75.69)	10(24.31)	4191.57)
Heard about family planning from the health professional			
No	2,077(90.39)	221(9.61)	2,298(87.27)
Yes	202(60.47)	133(39.53)	335(12.73)
Heard family planning by traditional communicators			
No	2,147(87.02)	321(12.98)	2,468(93.73)
Yes	132(79.95)	33(20.05)	165(6.27)
Heard about family planning from friends/relatives			
No	1,097(86.74)	168(13.26)	1,265(48.05)
Yes	1,182(86.43)	186(13.57)	1,368(51.95)
Covered by health insurance			
No	2,227(86.40)	351(13.60)	2,578(97.90)
Yes	53(95.08)	3(4.92)	55(2.10)
Age at first sex			
Never	2,129(100.0)	0(0.0)	2,129(80.88)
12–14	12(21.18)	46(78.82)	58(2.19)
15–16	99(28.20)	253(71.80)	352(13.38)
18–19	38(41.13)	55(58.87)	93(3.55)
Place of residence			
Urban	1,693(89.05)	208(10.95)	1,901(72.20)
Rural	587(80.16)	145(19.84)	732(27.80)
Region			
Banjul	31(89.89)	4(10.11)	35(1.32)
knifing	471(88.01)	64(11.99)	535(20.31)
Brikama	1,065(90.91)	107(9.09)	1,174(44.60)
mansakonko	81(82.39)	17(17.61)	98(3.73)
kerewan	221(85.27)	38(14.73)	259(9.84)
kuntaur	92(71.55)	37(28.45)	129(4.89)
janjanbureh	110(77.56)	32(22.44)	142(5.40)
basse	206(78.90)	55(21.10)	261(9.90)

### Factors associated with early pregnancy and motherhood

After adjusting the possible confounders, plenty of essential determinant variables were statistically significant for pregnancy and early motherhood. A unit increase in participant's age will result (AOR = 2.15, (CI 95%: (1.93,2.39)) times of high risk to have pregnancy early motherhood. In contrast, participants' wealth status and educational attainment were reversely associated with teenage pregnancy and early motherhood. Participants classified under the rich category (AOR = 0.35, (CI 95%: (0.23,0.54)) compared to the poor, having primary education (AOR = 0.58, (CI 95%: (0.43,0.79)) and secondary/higher level of education (AOR = 0.12, (CI 95%: (0.09,0.17)) times less likelihood of experiencing teenage pregnancy an early motherhood risk respectively. Regarding knowledge of the ovulatory cycle, those participants who realized their ovulatory cycle after their period ended have shown (AOR = 1.99, (CI 95%, (1.23,3.22)) times to become exposed to the risk of teenage pregnancy and early motherhood compared to their counterparts who knew ovulatory cycle. A girl from a family with more than ten members has (AOR = 1.25(CI, 95%: 1.01,1.55)) a chance of getting early pregnancy and motherhood than a girl who is a member of a family with less than ten (Table 2).

**Table 2:** Factors associated with early pregnancy and motherhood among 15–19 years old adolescents in the Gambia, GDHS 2019–20(*n* = 2,633)

Variables	Pregnancy and early motherhood		COR (95%, CI)	AOR (95%, CI
	No, n (%)	Yes, n (%)		
Age^b^(mean with SD^1^)	16.99(1.41)		2.05(1.87,2.25)	2.15(1.93,2.39)^b^
Wealth index				
Poor	759(80.00)	190(20.00)	1	1
Middle	432(82.96)	89(17.04)	0.79(0.61,1.02)	0.84(0.58,1.21)
Rich	1,088(93.57)	75(6.43)	0.27(0.19,0.36)	0.35(0.23,0.54)^b^
Educational attainment				
No education	279(64.22)	156(35.78)	1	1
Primary	374(79.52)	96(20.48)	0.49(0.38,0.64)	0.58(0.43,0.79)^b^
Secondary and above	1,626(94.13)	102(5.87)	0.13(0.09,0.17)	0.12(0.09,0.17)^b^
Occupational status				
Currently not working	1,665(88.68)	213(11.32)	1	1
Currently working	614(81.35)	141(18.65)	1.88(1.53, 2.31)	1.01(0.77,1.30)
Knowledge of ovulatory cycle				
During her period	90(89.02)	11(10.98)	1.36(0.72,2.59)	1.09(0.52,2.32)
After period ended	638(80.33)	156(19.67)	2.45(1.62,3.72)	1.99(1.23,3.22)^b^
Middle of the cycle	332(84.51)	61(15.49)	1.51(0.93,2.45)	1.55(0.88,2.71)
Before period begin	306(87.90)	42(12.10)	1.51(0.93,2.46)	1.54(0.91,2.79)
At anytime	196(89.09)	24(10.91)	1	1
Do not know	717(92.40)	59(7.60)	0.76(0.48,1.21)	0.74(0.44,1.23)
Knowledge of any methods				
No	127(92.52)	10(7.48)	1	1
Yes	2,153(86.25)	343(13.75)	2.04(1.14,3.64)	1.71(0.88,3.33)
Household member				
1–10	1,055(87.24)	154(12.76)	1	1
10 +	1,224(86.01)	199(13.99)	1.27(1.03,1.56)	1.25(1.01,1.55)^b^
Owning mobile phone				
No	1,106(89.20)	134(10.80)	1	1
Yes	1,173(84.24)	219(15.76)	0.74(0.59,0.91)	0.82(0.61,1.11)
Use of the internet				
Never	1,140(88.07)	154(11.93)	1	1
Ever use	1,140(85.14)	199(14.86)	1.27(1.04,1.56)	1.21(0.89,1.66)
Watching to television				
No	958(83.65)	187(16.35)	1	1
Yes	1,322(88.83)	166(11.17)	1.68(1.37,2.07)	0.90(.69,1.17)
Covered by health insurance				
No	2,227(86.40)	351(13.60)	1	1
Yes	53(95.08)	3(4.92)	3.67(0.88,15.31)	1.09(0.24,5.02)
Place of residence				
Urban	1,693(89.05)	208(10.95)	1	1
Rural	587(80.16)	145(19.84)	0.48(0.39, 0.60)	0.77(0.55,1.09)

Where ^a^ represents continues variables and ^b^ is significant variables at *p*-value of < 0.05, *SD*^*1*^ Standard deviation

## Discussion

Based on the most recent Gambian demographic and health survey data, they aimed to assess the current national-level prevalence and identify hindering factors of teenage pregnancy and early motherhood among late female adolescents in the Gambia. The overall burden of pregnancy and early motherhood was discovered by 13.42% (12.17, 14.78). This finding is lower than studies done in Pakistan(42.5%) [40], Latin America (19.2%) [41], and Nigeria(22.9%) [42], meanwhile it is almost in agreement with studies done in South Africa (11.0%) [43]. However, it is higher than a systematic review done in Africa, northern Africa regions at 9.2% [44], and Ethiopia at 7.7% [45]. The variation might be due to differences in knowledge sociodemographic and socioeconomic dimensions and religious and cultural outlooks of early marriage. Another possible reason might be differences in the number of adolescents involved in the study and the geographical distribution of teenagers. In developed nations, most adolescents are the victim of pornography movie exposure, which is associated with premarital teenage pregnancy [46]. In addition to the individual level factors of education, attitude, analyzed number, countries profiles, and accomplishments in pregnancy and early motherhood might affect the overall burden.

The study discovered that a unit increase in age leads late-adolescent girls to have an early pregnancy and motherhood. Other literature has concluded the same finding with the current study in Nigeria [47] and Ethiopia [19]. This might be because sexual maturation reaches its peak given as age increases due to; for these reasons, adolescent sexual curiosity leads to exposure to pornography, participation in sexual activities, and increased vulnerability to sexual abuse [48]. On the other hand, in addition to participants' expression of sexual behaviours and tendencies to have sexual life, another external pressure from the parents and other relatives perhaps increases the risk of having marriage due to the parent's belief that the girl is old enough to get married. For the sake of their morale and the culture of the society, they can force girls with the idea of marrying and to prevent giving birth to a girl out of wedlock [24, 49, 50].

Participants classified under the rich category have a less likelihood of a positive tendency to experience early pregnancy and motherhood than poor. This is similar to studies done in Nigeria [42] and Ethiopia [51]. Studies done in Africa and the United States of America have shown that low-income girls may consider early marriage and sex as a means of income generation to lead their daily lives with money scarcity [13, 52–54].

The other crucial variable which identified significant statistically was education. The study declared that participants with primary and higher educational attainment had shown less likely association to have early pregnancy and early motherhood than those who did not have formal educational attainment. This finding is in agreement with studies done in various places in Africa [44], Pakistan [40], Nepal [55], and Ethiopia [51]. This is conceivably due to more well-educated girls may have better knowledge and attitudes about the potential health and lifestyle situations than uneducated girls through school, the internet, mass media, and books [55, 56]. With the help of better knowledge, they will be better able to prevent unwanted pregnancies and develop better plenty of preventive strategies.

Furthermore, girls who attend school for ten years marry six years later, as schooling increases autonomy, decision-making, and economic independence, causing the marriage to be postponed. Regarding g to the benefit of education on the prevention of pregnancy and early motherhood, a study stated that each additional year of schooling results in a 10% decrease in fertility and a 10% increase in contraception use [57]. These girls have more probability of realizing what type of prevention mechanism they should take, from abstinence to proper utilization of preventive pregnancy. For instance, studies done in Malawi and Kenya have revealed that the majority of sexually active teenage did not prevent pregnancy unless they used modern contraceptive methods. From here, one can understand that being exposed to training and education related to reproductive health concerns could dramatically reduce teenagers being pregnant and early motherhood.

An important variable that was one of the determinants for the outcome variable was the participants' knowledge of the ovulatory cycle; a girl who knows after the ovulatory cycle is more likely to become pregnant and become a mother than a participant who knows about her ovulatory process. This study is in agreement with the survey done in Africa [58], Ethiopia [51], and Ghana [59]. This is perhaps explained by a girl who is aware of her ovulatory cycle date of starting and ending is less likely to become pregnant. This is because a girl who is well aware of her ovulatory cycle can have sex only during the days when she is less likely to get pregnant, and perhaps she uses condoms pills and counts of ovulatory days.

Additionally, in line with findings from earlier literature, this study discovered that the likelihood of early pregnancy and motherhood increased with family size. Early pregnancies in the Philippines Indonesia, Rwanda, and Nigeria showed that large families (those with more than 10 family members) were more than twice as common as smaller numbers of household members [60–63]. As the number of family members increases, parents may choose to have their children work or live independently outside the home due to economic constraints. In other words, as the number of children increases, the risk of withdrawal from school, sexual intercourse exposure and pregnancy increases as girls may want to be outside the home of their own volition or become economically self-sufficient. Studies revealed that teenage females may be exposed to their parents' sexual behaviours too early in a small family setting, which later encourages their sexual interest and behaviour [64, 65]. The study comes to the conclusion that homes with parents who get along well and set clear boundaries for their children, discipline them, and communicate against pregnancy help reduce the number of teenage pregnancies and motherhood.

## Conclusion and recommendation

This study concluded that pregnancy and early motherhood burdens among late adolescent girls are comparatively high in the Gambia. The study found that the age of girls, wealth status, educational attainment, and knowledge of ovulatory cycle have shown statistically significant associations with the outcome variable of pregnancy and early motherhood in the Gambia. Better results can be achieved by empowering females’ power, strengthening girls' participation in education, paying attention to girls in need of finance, and providing life skills and reproductive health issues training.

## Data Availability

All data concerning this study are accommodated and presented in this document.
